# Depot-specific adipocyte-extracellular matrix metabolic crosstalk in murine obesity

**DOI:** 10.1080/21623945.2020.1749500

**Published:** 2020-04-09

**Authors:** Clarissa Strieder-Barboza, Nicki A. Baker, Carmen G. Flesher, Monita Karmakar, Ayush Patel, Carey N. Lumeng, Robert W. O’Rourke

**Affiliations:** a Department of Surgery, University of Michigan Medical School, Ann Arbor, MI, USA; b Department of Pediatrics and Communicable Diseases, University of Michigan Medical School, Ann Arbor, MI, USA; c Undergraduate Research Opportunity Program, University of Michigan, Ann Arbor, MI, USA; d Graduate Program in Immunology, University of Michigan, Ann Arbor, MI, USA; e Graduate Program in Cellular and Molecular Biology, University of Michigan, Ann Arbor, MI, USA; f Department of Surgery, Veterans Affairs Ann Arbor Healthcare System, Ann Arbor, MI, USA

**Keywords:** Adipocyte, extracellular matrix, depot, visceral, subcutaneous, adipose tissue, obesity, adipogenesis, insulin resistance, glucose uptake

## Abstract

Subcutaneous (SAT) and visceral (VAT) adipose tissues have distinct metabolic phenotypes. We hypothesized that the extracellular matrix (ECM) regulates depot-specific differences in adipocyte metabolic function in murine obesity. VAT and SAT preadipocytes from lean or obese mice were subject to adipogenic differentiation in standard 2D culture on plastic tissue culture plates or in 3D culture in ECM, followed by metabolic profiling. Adipocytes from VAT relative to SAT manifested impaired insulin-stimulated glucose uptake and decreased adipogenic capacity. In 3D-ECM-adipocyte culture, ECM regulated adipocyte metabolism in a depot-specific manner, with SAT ECM rescuing defects in glucose uptake and adipogenic gene expression in VAT adipocytes, while VAT ECM impaired adipogenic gene expression in SAT adipocytes. These findings demonstrate that ECM-adipocyte crosstalk regulates depot-specific differences in adipocyte metabolic dysfunction in murine obesity.

## Introduction

Visceral adipose tissue (VAT, approximated by epididymal adipose tissue in mice), is more strongly associated with metabolic disease in mice and humans than subcutaneous adipose tissue (SAT, approximated by inguinal subcutaneous adipose tissue in mice) [1,2]. While significant data implicate intrinsic differences in adipocyte cellular function in depot-specific functionality [3–5], other components of the local adipose tissue microenvironment are altered in response to over-nutrition and may contribute to the distinct metabolic profiles of VAT and SAT. Supporting this concept, a recent study reported that donor preadipocytes isolated from SAT or VAT depots behave in a manner consistent with the recipient anatomic injection site rather than their depot of origin during high-fat feeding, suggesting that the adipose tissue microenvironment regulates depot-specific adipocyte function [6]. The extracellular matrix (ECM) is a primary constituent of adipose tissue microenvironment and alterations in adipose tissue ECM are associated with both murine and human obesity and metabolic disease [7,8]. The adipose tissue ECM manifests quantitative and qualitative changes in obesity, with a preponderance of data demonstrating increased ECM deposition as well as alterations in ECM morphology and expression of specific ECM constituents [9–11]. Despite these observations, the specific role of the ECM in regulating adipose tissue metabolic function remains poorly defined. Our group recently demonstrated disease-specific metabolic regulation of human adipocytes by adipose tissue ECM, suggesting that the ECM modulates adipocyte insulin responses and lipolysis in human metabolic disease [12]. In the present study, we hypothesized that ECM regulates adipocyte cellular metabolism in a depot-specific manner in murine obesity, such that SAT ECM induces a beneficial metabolic phenotype in VAT adipocytes, while VAT ECM impairs SAT adipocyte function. We tested this hypothesis using a 3D-ECM-adipocyte culture system that permits the study of depot-specific ECM-adipocyte crosstalk.

## Materials and Methods

### Mice

Experiments were approved by the University of Michigan Institutional Animal Care and Use Committee consistent with AAALAC regulations. Eight-week-old male C57Bl/6 mice were fed normal diet (ND) or high-fat diet (HFD) (60% fat calories, Research Diets Inc., Cat#D12492) for 8 weeks. The number of mice used for each experiment is described in the figures. Body weights were measured weekly. At the end of the feeding protocol, a glucose tolerance test (GTT) was performed as described [13], followed by euthanasia and collection of VAT (epididymal fat pad) and SAT (inguinal/flank fat pad).

### Cell culture

The stromal vascular cell fraction was isolated from VAT and SAT using type II collagenase digestion. Preadipocytes were isolated by plate adherence after two consecutive passages, expanded to confluence, then frozen and stored in liquid nitrogen until use. For *2D-plastic culture*, preadipocytes were thawed and plated on standard plastic tissue culture plates and subjected to *in vitro* adipogenic differentiation for 14 days in an adipogenic medium composed by DMEM:F12 50:50, 10% foetal bovine serum (FBS; Gibco|ThermoFisher Scientific Inc., Cat# 26140), 1% antimycotic-antibiotic solution (ThermoFisher Scientific Inc, Cat#15240062), and the following reagents from Sigma-Aldrich Inc.: 10 mg/l transferrin (Cat# 10652202001), 33 µM biotin (Cat#B0301), 0.5 µM human insulin (Cat# 91077 C), 17 µM pantothenate (Cat# C8731), 100 nM dexamethasone (Cat# D9184), 2 nM 3,3ʹ,5-Triiodo-L-thyronine sodium salt (Cat#T6397), 1 µM ciglitazone (Cat#230950), 540 µM Isobutyl-1-methylxanthine (Cat#I7018), as previously described [12]. For *3D-ECM culture*, SAT and VAT ECM were isolated from mice fed HFD for 8 weeks following a protocol described by our laboratory [14]. After ECM isolation, 3D-ECM/adipocyte cultures were constructed: ECM was rinsed in 70% EtOH, rehydrated in PBS, cut into 100 mg fragments, and seeded with 60 000 preadipocytes in 20 µl of 10% FBS DMEM:F12 in 24-well plates, testing depot-matched (ECM/preadipocytes: VAT/VAT, SAT/SAT) and depot-mismatched (VAT/SAT, SAT/VAT) combinations. ECM/preadipocytes were incubated at 37°C, 5% CO_2_ for 40 min to allow cells to adhere before adding 0.5 ml of 10% FBS DMEM:F12 to each well. After 24 h incubation at 37° C, 5% CO_2_, ECM-preadipocytes were transferred to a fresh 24-well culture plate containing 0.5 ml of 10% FBS DMEM:F12/well, cultured for 72 h, followed by culture in adipogenic medium for 14 days, as described for 2D-plastic culture, to generate mature adipocytes [12].

### In vitro *metabolic assays*

#### Adipocyte area

VAT and SAT adipocyte area (µm^2^) was measured using fixed hematoxylin/eosin-stained sections imaged on an Olympus IX-81 fluorescent microscope using Texas Red channel (595–605 nM). Images were captured as multiple TIFF-grey-scale images and analysed with ImageJ software. Adipocyte area was measured in 200–500 cells from multiple slides per specimen and averaged for each tissue sample.

#### Lipid accumulation

Scanning electron microscopy [12] and Oil Red-O staining (Sigma Aldrich Inc., Cat# O0625) were used to image 3D-ECM/adipocytes to assess lipid accumulation after differentiation. AdipoRed (Lonza Inc., Cat# PT-7009) was used to quantify adipocyte lipid accumulation in 2D-plastic cultures and standardized by total protein as measured by Pierce BCA assay (ThermoFisher Scientific Inc., Cat# 23225).

#### Quantitative PCR (qPCR)

RNA from SAT and VAT was extracted in Trizol and adipocytes from 2D and 3D cultures using RNeasy Mini Kit and RNeasy Fibrous Tissue MiniKit (Qiagen Inc., Cat# 74104, 74704), respectively. Purity, concentration, and integrity of mRNA were evaluated using a NanoDrop 1000 spectrophotometer (ThermoFisher Scientific Inc., Cat# ND-ONE-W). Of note, RNA extraction from ‘empty ECM’ not seeded with cells yielded no detectable or amplifiable RNA, confirming complete removal of RNA from ECM preparations prior to seeding with cells. Equal amounts of RNA from whole SAT and VAT or adipocytes from 2D and 3D culture systems were reverse-transcribed using a High Capacity cDNA Archive Kit (Applied Biosystems Inc., Cat# 4368814). cDNA was studied with qPCR using Taqman gene expression assays and reagents (Life Technologies Inc.) using a StepOnePlus thermocycler (Applied Biosystems Inc., Cat# 4376600). Data are presented as fold changes calculated from least-squares mean differences according to the 2^−ΔΔCT^ method [15] and normalized to the mean of β2-microglobulin (B2 M) housekeeping gene, for which CT values did not vary between adipose depots from HFD mice, nor between the different ECM-adipocyte matches (P > 0.05). A CT value of 40 was assigned to undermined CT values for fold change calculations.

#### Glucose uptake assay (GUA)

Insulin responses were evaluated with GUA as described [12]. Briefly, adipocytes in 2D-plastic or 3D-ECM cultures were serum-starved overnight then incubated with PBS/2% BSA at 37°C for 2 h, followed by insulin stimulation (200 nM) 37°C for 40 min. Glucose uptake was assessed by uptake of 2-deoxy glucose (0.1 mM; Sigma-Aldrich Inc., Cat#D6134) and deoxy-d-glucose-2-[1,2–3 H(N)] (2μCi/ml; PerkinElmer Inc., Cat#NET549001MC) for 40 min, and measured with scintillation counting (counts per minute, cpm) normalized to cell protein (DC™ Protein Assay, Bio-Rad, Inc., Cat#5000112).

### Statistical analysis

Statistical analysis was performed in STATA-version 15 (StataCorp LLC). A sample size calculation was done using GUA data from a pilot study with similar 3D-ECM/adipocyte cultures from four mice. Sample size analysis revealed the need for a total of 16 mice to observe similar differences at an alpha of 5% in order to achieve 90% power. We assumed that the correlation between the timepoints would be 0.2 and inflated our numbers to account for 20% dropout rate. We planned to include 20 mice in our study. In vivo metabolic data (body weight and GTT) was analysed with a one-way repeated-measures ANOVA followed by Tukey multiple comparisons analysis and area under the curve (AUC) was calculated using the trapezoidal method. Data comparing VAT vs. SAT in ND vs. HFD mice were analysed by a two-way ANOVA followed by Tukey multiple comparisons analysis. In vitro, GUA and qPCR data were analysed by a two-way repeated-measures ANOVA to analyse the effect of treatment with VAT/SAT ECM on the adipocytes extracted from the two different depots (VAT/SAT) from the same mice. Separate models were used to analyse the effect of the ECM treatment on basal and insulin-stimulated adipocyte glucose uptake. Post hoc pairwise comparisons were performed using Bonferroni’s adjustment for multiple comparison. Figures display means with error bars representing standard errors of the mean.

## Results

### Obesity induces systemic insulin resistance and alters adipose tissue ECM gene expression profile

**Figure 1.**
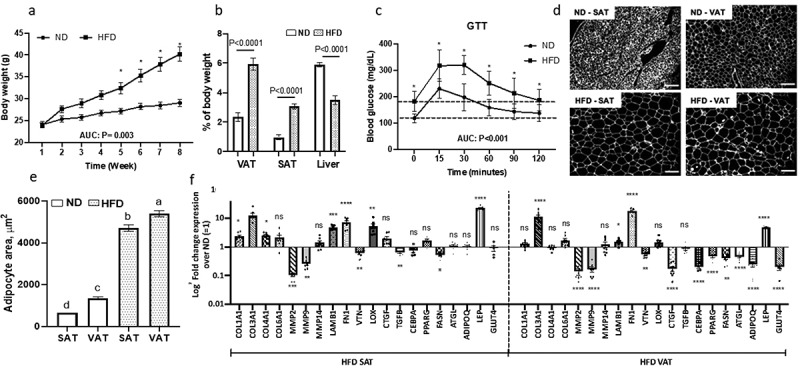
Systemic insulin sensitivity and adipose tissue ECM and adipogenic gene expression are altered in obesity. (a) Weekly body weights of mice fed ND or HFD for 8-weeks. *P < 0.001 comparing ND and HFD arms. (b) Organ weights and (c) glucose tolerance test (GTT) performed at end of 8-week of ND or HFD (interaction diet vs. time during GTT: P = 0.044). Area under curve (AUC) for body weight and GTT was calculated by trapezoidal method. (d) Representative fluorescence images of sectioned adipose tissues (scale bars: 100 µm) used for (e) quantifying adipocyte area. Different letters (a-d) represent P ≤ 0.05 using Tukey multiple comparisons analysis. (f) ECM and adipogenic gene expression profile in adipose tissues measured with qPCR, displayed as fold change in transcript levels in VAT and SAT from HFD mice relative to matched tissues from ND mice as referent = 1. Statistical analysis was performed in delta CT values. ns: P > 0.05, *P ≤ 0.05, **P < 0.01, ***P < 0.001, ****P < 0.0001 comparing delta CT values for VAT or SAT in HFD relative to ND mice; n = 8 ND, 8 HFD mice

An 8-week HFD caused increased body weight by 5.30 ± 1.35 g (mean ± SEM; P = 0.001, Figure 1(a)), increased VAT and SAT depot weights, decreased liver mass (Figure 1(a,b)), and systemic insulin resistance as measured by GTT (Figure 1(c)). Compared with ND, HFD induced adipocyte hypertrophy that was greater in VAT than SAT (Figure 1(e)), and depot-specific alterations in adipose tissue expression of multiple ECM and adipogenic genes (Figure 1(f)): *COL1A1, COL4A1, and LOX* were upregulated in SAT, while *COL3A1, LAMB1* and *FN1* were upregulated in both, VAT and SAT; *MMP2, MMP9*, and *VTN* were downregulated in SAT and VAT; *CTGF* expression was increased in SAT but decreased in VAT. The adipogenic genes *CEBPA, PPARG, FASN, ATGL, ADIPOQ*, and *GLUT4* were downregulated in VAT but not SAT in response to HFD, while *LEP* expression was increased in both depots in response to HFD. These data demonstrate that obesity impairs systemic insulin sensitivity, induces adipocyte tissue hypertrophy, and induces a gene expression pattern consistent with increased ECM deposition in both depots, and decreased adipogenesis in VAT but not SAT.

### Obesity regulates adipocyte metabolic function in a depot-specific manner

**Figure 2.**
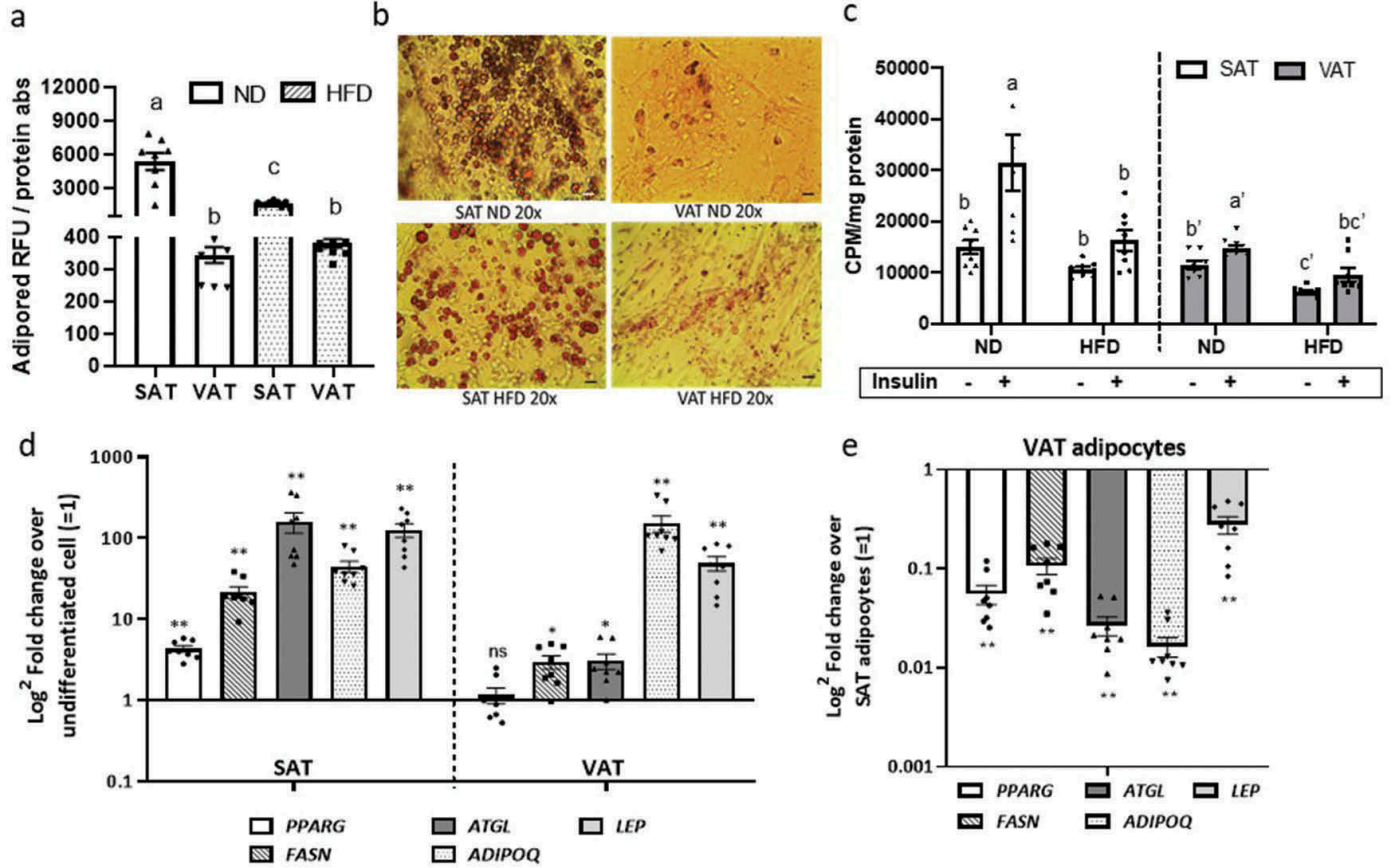
Depot-specific differences in adipocyte metabolism in response to obesity. Effect of diet and adipose tissue depot on adipogenic differentiation of SAT and VAT adipocytes after 14d of *in vitro* differentiation in 2D culture demonstrated by lipid accumulation measured with (a) AdipoRed (Lonza) and (b) Oil Red-O staining (scale bars: 100 µm). Different letters (a-c) represent P ≤ 0.05 using Tukey multiple comparisons analysis. (c) Effect of diet on basal and insulin-stimulated glucose uptake in SAT and VAT 2D adipocytes measured with ^3^ H-2-deoxy-glucose uptake assay. Different letters (a-d) represent P ≤ 0.05 for Tukey multiple comparisons analysis. Statistical analysis for SAT and VAT adipocytes were performed separately. Insulin-stimulated glucose uptake was higher in ND SAT than ND VAT (P < 0.0001), but similar between HFD SAT and HFD VAT (P = 0.395; not indicated on graph). (d,e) Effect of obesity on gene expression of adipogenic and mature adipocyte markers of 2D adipocytes measured by qPCR. In (d), values are shown as log^2^ fold change in adipocyte gene expression relative to matched undifferentiated preadipocytes as a referent = 1. In (e), values are shown as log^2^ fold change in VAT adipocytes relative to matched SAT adipocytes as a referent = 1. *P < 0.01, **P < 0.0001 using unpaired t-tests comparing delta CT values between VAT and SAT arms; n = 8 ND, 8 HFD mice

To evaluate adipocyte cellular metabolic responses to obesity, we studied adipogenesis and glucose uptake in adipocytes differentiated *in vitro* from preadipocytes in 2D-plastic culture. Adipogenesis in both VAT and SAT adipocytes was decreased in response to HFD compared to ND (Figure 2(a)), as demonstrated by decreased Adipored (Figure 2(b)) and Oil Red-O staining (Figure 2(c)), consistent with multiple prior data [5,16–18]. Adipose tissue depot influenced adipocyte glucose uptake independent of obesity, with basal and insulin-stimulated glucose uptake decreased in VAT relative to SAT adipocytes in both ND and HFD states. HFD-induced obesity decreased basal and insulin-stimulated glucose uptake in VAT and SAT adipocytes relative to cells from ND animals (Figure 2(c)). In mice fed HFD, both VAT and SAT-derived preadipocytes successfully differentiated into adipocytes as demonstrated by increased gene expression of adipogenic and mature adipocyte markers relative to undifferentiated cells (Figure 2(d)). However, VAT adipocytes, relative to SAT adipocytes, had decreased adipogenesis as demonstrated by lower expression of *PPARg, FASN, ATGL, ADIPOQ*, and *LEP* (Figure 2(e)). These data demonstrate depot-specific defects in adipogenesis and adipocyte cellular insulin sensitivity that are amplified by HFD.

### Adipose tissue ECM regulates adipocyte metabolism in a depot-specific manner

**Figure 3.**
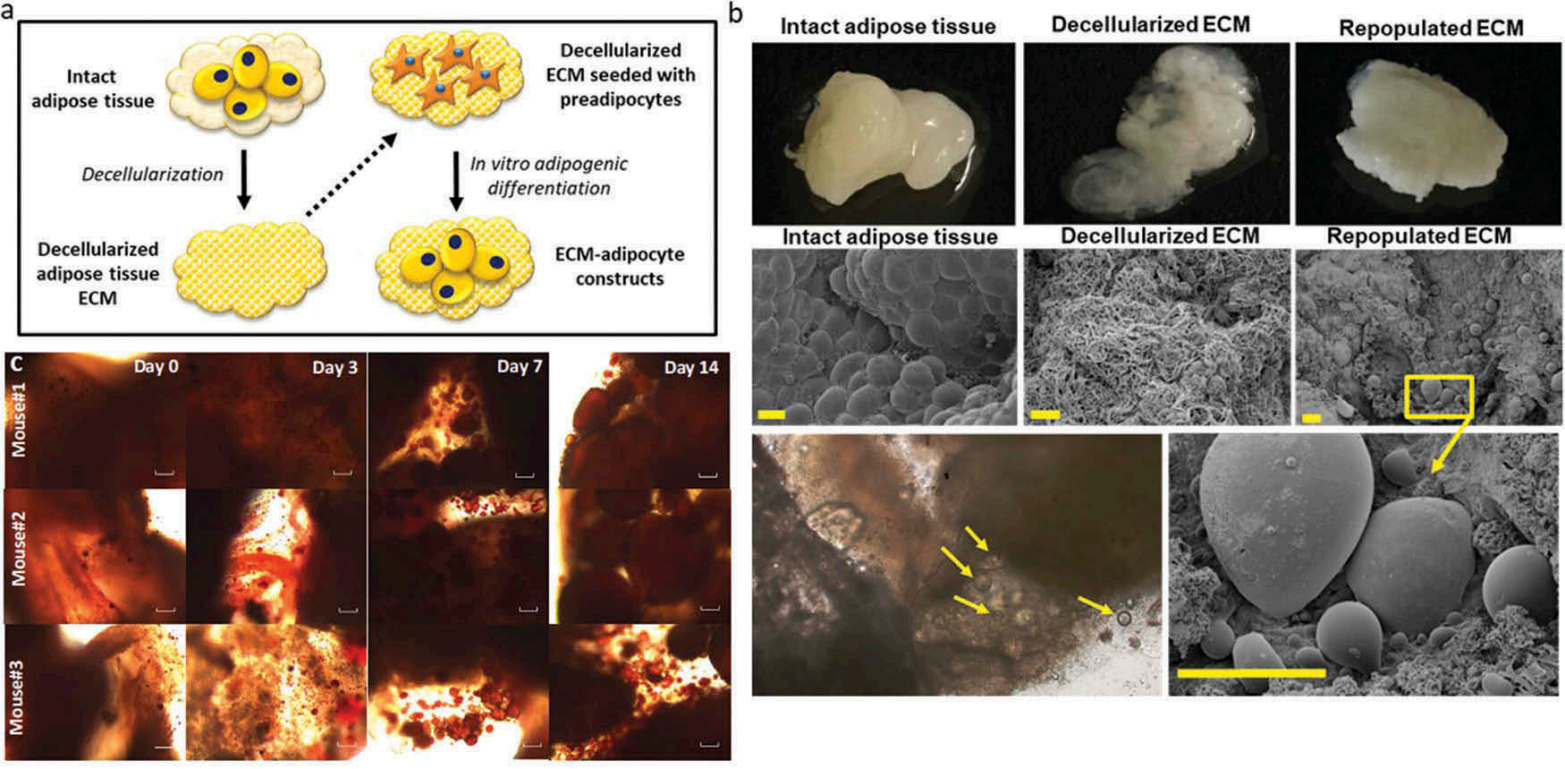
Differentiation of adipocytes in a 3D ECM-adipocyte *in vitro* culture model. (a) Graphic representation of ECM-adipocyte co-culture system. (b) Photographs, scanning electron micrographs of whole VAT, decellularized VAT, and decellularized VAT ECM repopulated with VAT preadipocytes and differentiated 14 days. Scale bars: 100 µm for all images, except decellularized ECM (10 µm). (c) Light microscopy images of intact VAT 3D-ECM/adipocyte culture (*n* = 3 male 16-week old C57BL6 mice fed ND) stained with Oil Red-O on different time points during adipogenic differentiation. Images were obtained with a 10X objective on an Olympus CKX41 microscope with Infinity 1 camera and Lumenera software (scale bars: 100 µm)

**Figure 4.**
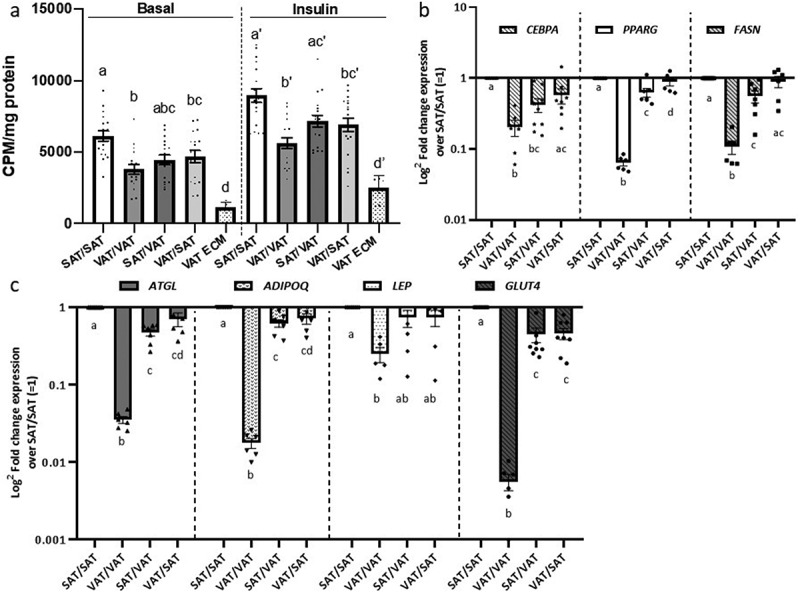
Depot-specific murine ECM-adipocyte metabolic crosstalk (a) Basal and insulin-stimulated (200 nM, 40 min) glucose uptake in combinations of VAT and SAT 3D ECM/adipocytes cultures. Data bars labelled with depot source (VAT, SAT) of ECM and adipocytes (ECM/AD); for example, VAT/VAT denotes both ECM and preadipocytes derived from VAT, while VAT/SAT denotes ECM from VAT combined and preadipocytes from SAT. Empty VAT ECM samples were included as negative controls (n = 5). Separate models were used to analyse the effect of the ECM treatment in systems with and without insulin in glucose uptake assays (as indicated by vertical discontinued bar and apostrophes). Different letters (a-d) indicate P ≤ 0.05 in post hoc pairwise comparisons using Bonferroni’s adjustment for multiple comparison (ECM/adipocytes from n = 18 HFD mice). (b-c) Gene expression of adipogenic (b) and mature adipocyte markers (c) in ECM/adipocyte cultures measured with qPCR. Values are displayed as log^2^ fold change in gene expression relative in each arm relative to the referent arm of SAT/SAT ECM/adipocytes = 1. Different letters (a-d) indicate P ≤ 0.05 in post hoc pairwise comparisons using Bonferroni’s adjustment for multiple comparisons (ECM/adipocytes from n = 9 HFD mice)

To investigate the role of ECM in regulating adipocyte cellular metabolism in obesity, we used a 3D-ECM/adipocyte co-culture model comprised of tissue constituents from distinct depots, as previously reported by our laboratory [12] (Figure 3(a)). Using tissues from HFD-fed mice, we examined metabolic responses of adipocytes differentiated in decellularized ECM, studying all combinations of ECM/adipocytes (VAT/VAT, SAT/SAT, VAT/SAT, SAT/VAT). Preadipocytes seeded into ECM were differentiated into mature adipocytes that appeared to contain unilocular lipid droplets, as evidenced by scanning electron microscopy (Figure 3(b)) and standard light microscopy of Oil Red O stained 3D-ECM/adipocyte cultures (Figure 3(c)). VAT/VAT ECM-adipocyte cultures, relative to SAT/SAT cultures, demonstrated decreased glucose uptake, recapitulating depot-specific differences in glucose uptake observed in adipocytes cultured in isolation in standard 2D-plastic culture. Importantly, differentiation of VAT adipocytes in SAT ECM (SAT/VAT) increased insulin-stimulated glucose uptake to levels similar to SAT/SAT ECM-adipocyte constructs, consistent with a rescue effect of SAT ECM on VAT adipocyte metabolism. Conversely, VAT ECM impaired glucose uptake in SAT adipocytes relative to SAT adipocytes cultured in SAT ECM (Figure 4(a)). We also studied the regulation of adipogenic genes in ECM-adipocyte cultures using qPCR (Figure 4(b,c)). Adipogenic gene expression in mature adipocytes was decreased in VAT/VAT ECM-adipocyte cultures relative to SAT/SAT cultures, consistent with decreased VAT adipogenesis observed in 2D adipocyte culture. Importantly, expression of the adipogenic genes *PPARG, FASN, ADIPOQ*, and *ATGL*, but not *LEP*, were increased in VAT adipocytes differentiated in SAT ECM relative to VAT adipocytes differentiated in VAT ECM, consistent with a rescue effect of SAT ECM on VAT adipocytes with respect to adipogenesis. Conversely, an impairment effect on the expression of *ADIPQ* and *ATGL*, but not *PPARG, FASN*, or *LEP*, were observed on SAT adipocytes differentiated in VAT ECM (Figure 4(b,c)). Together, these data demonstrate depot-specific rescue and impairment effects of adipose tissue ECM on adipogenesis and adipocyte cellular insulin sensitivity.

## Discussion

Subcutaneous and visceral adipose tissue depots manifest divergent ontogeny, metabolic phenotype, and disease associations, but mechanisms underlying these differences remain poorly understood. We recently demonstrated disease-specific regulation of adipocyte cellular metabolism by the ECM in humans, such that non-diabetic adipose tissue ECM was capable of rescuing impaired metabolic function in diabetic adipocytes [12]. In the present study, we hypothesized that similar ECM-adipocyte crosstalk may underlie depot-specific differences in adipose tissue metabolic phenotype in murine obesity.

We first demonstrated depot-specific differences in cellular metabolic phenotype in murine adipocytes cultured in isolation in 2D-plastic culture, including defects in glucose uptake in VAT compared to SAT adipocytes in both ND and HFD conditions, with exacerbation of this phenotype in HFD. These findings are consistent with prior human and murine data supporting increased cellular insulin resistance in VAT relative to SAT in obese and non-obese states [12,19,20]. We also demonstrate increased hypertrophy in VAT relative to SAT adipocytes in the non-obese state that is markedly increased in both depots in obesity, consistent with multiple prior data [21]. We demonstrate decreased glucose uptake in VAT relative to SAT adipocytes in mice and humans, consistent with some [12,22] but not all [23,24] prior published data. Differences in the methodology used to measure glucose uptake as well as species-specific differences may account for these conflicting data. Finally, VAT adipocytes manifest markedly decreased adipogenic potential relative to SAT adipocytes, also consistent with multiple prior data [5,16–18]. Together our findings support relative impairment of murine adipocyte cellular insulin resistance in VAT compared to SAT adipocytes, defects that are amplified in the obese state.

Previous data demonstrate alterations in adipose tissue ECM in obesity [7,8]. We demonstrate that 8-week HFD induces changes in ECM-related gene expression in VAT and SAT, including increased collagen expression and decreased MMP expression consistent with a ‘pro-fibrotic’ state. These observations led us to study murine ECM-adipocyte metabolic crosstalk using a 3D-ECM/adipocyte co-culture model that allows dissection of the individual roles of ECM and adipocytes from different depots in determining ultimate tissue metabolic phenotype. In this model, ECM supports adipogenic differentiation of preadipocytes, with development of unilocular lipid droplets in mature adipocytes, in contrast to the multilocular lipid droplet adipocyte phenotype seen in standard 2D-plastic culture. Our study and others [25–27] suggest that ECM and other 3D culture systems provide a more physiologic environment for adipocyte differentiation than standard 2D plastic culture. Our findings also demonstrate that the ECM contributes to depot-specific differences in global tissue metabolism, with SAT-derived ECM demonstrating the capacity to rescue defects in glucose uptake in VAT adipocytes, while conversely, VAT ECM impaired glucose uptake in SAT adipocytes. We observed a similar depot-specific rescue effect of SAT ECM on the expression of a subset of adipogenic genes in VAT adipocytes, as well as an impairment effect of VAT ECM on SAT adipocytes on the expression of a different subset of adipogenic genes. Together, these data demonstrate distinct effects of ECM on different aspects of adipocyte metabolism and suggest that the effects of ECM on adipocyte cellular metabolism may in part be regulated via effects on adipogenesis. Others have shown that culture of murine adipocytes in 3D collagen hydrogels similarly rescues defects in VAT adipocyte adipogenesis [25], corroborating our data, which demonstrate a similar effect in a more physiologic ECM substrate. Of note, in our prior study of human diabetes-specific ECM-adipocyte crosstalk, adipogenic gene expression was not regulated by the ECM in a diabetes-specific manner [12], suggesting different mechanisms underlying disease- and depot-specific crosstalk in humans and mice.

Our prior work demonstrates diabetes-specific ECM-adipocyte metabolic crosstalk in humans [12], suggesting commonalities between mice and humans with respect to ECM-adipocyte crosstalk. Further research will be necessary to determine if ECM-adipocyte crosstalk is depot-specific in humans. We utilized an 8-week 60% fat diet which we have shown induces adipose tissue and systemic metabolic dysfunction [28]; different diet regimens may provide different results. The ECM-adipocyte culture model lacks multiple adipose tissue constituents, but this simplicity permits the isolated study of specific effects of ECM on adipocyte metabolism. This culture model provides a tractable system to study the role of other cell types (e.g. leukocytes and other immune cells, endothelial cells) in regulating diverse aspects of ECM-adipocyte crosstalk, research in progress. Finally, further research will be required to elucidate mechanisms underlying the observed ECM-adipocyte crosstalk, such as altered ECM composition (e.g. increased collagens, decreased MMP expression, increased advanced glycation end-products), as well as mechanical properties (e.g. elasticity) in different depots and obesity states, with the ultimate goal of identifying specific ECM mediators of adipocyte metabolism.

We describe a novel ECM-adipocyte culture system adapted to murine tissues that permits dissection of the functional roles of different adipose tissue constituents on ultimate tissue metabolic phenotype. We used this model system to demonstrate depot-specific ECM-adipocyte metabolic crosstalk in murine obesity, such that adipose tissue ECM is capable of reprogramming adipocyte metabolic phenotype, in part via regulation of adipogenesis. These data support a role for the ECM in regulating depot-specific differences in adipocyte cellular metabolism and suggest that the ECM contributes to depot-specific differences in adipocyte adipogenic potential.
